# Stemness and differentiation potential-recovery effects of sinapic acid against ultraviolet-A-induced damage through the regulation of p38 MAPK and NF-κB

**DOI:** 10.1038/s41598-017-01089-5

**Published:** 2017-04-19

**Authors:** Young Sun Hwang, See-Hyoung Park, Mingyeong Kang, Sae Woong Oh, Kwangseon Jung, Yong Seek Park, Jongsung Lee

**Affiliations:** 1 Department of Genetic Engineering, College of Biotechnology and Bioengineering, Sungkyunkwan University, Suwon City, 164-19 Gyunggi Do Republic of Korea; 2 Department of Dental Hygiene, College of Health Science, Eulji University, Seongnam City, 131-35 Gyunggi Do Republic of Korea; 3 Department of Bio and Chemical Engineering, Hongik University, 300-16 Sejong City, Republic of Korea; 4 Department of Microbiology, School of Medicine, Kyung Hee University, 024-53 Seoul, Republic of Korea; 5 Skincure Life Science Institute, Seongnam City, 132-16 Gyunggi Do Republic of Korea

## Abstract

Ultraviolet A (UVA) irradiation exerts negative effects on stemness and differentiation potential of stem cells. This study aimed to explore the effect of sinapic acid on UVA-irradiation-induced damages to stemness and differentiation potential of human-adipose-tissue-derived mesenchymal stem cells (hAMSCs) and its UVA-antagonist mechanisms. Sinapic acid attenuated UVA-induced reduction in the proliferative potential and stemness by upregulating OCT4, SOX2, and NANOG. In addition, sinapic acid significantly recovered UVA-induced reduction in expression level of hypoxia-inducible factor (HIF)-1α. The antagonist effect of sinapic acid against stemness damage was mediated by reduceing PGE_2_ production through inhibition of p38 MAPK and NF-κB. Moreover, sinapic acid attenuated UVA-induced reduction in differentiation potential by downregulating the expression of macrophage migration inhibitory factor (MIF) and Kruppel-like factor (KLF) 2 gene while activating AMP-activated protein kinase (AMPK). UVA-induced inhibition of adipogenic differentiation was mediated by reducing MIF production through suppression of NF-κB. Taken together, these findings suggest that sinapic acid may ameliorate UVA-irradiation-induced reduced stemness and differentiation potential of hAMSCs. Therefore, sinapic acid might have potential as an antagonist agent to attenuate damages caused by UVA.

## Introduction

Ultraviolet A (UVA) irradiation is either beneficial or harmful to various cell types and tissues^1–3^. A number of studies have shown that UVA irradiation affects the subcutis and its several cell types including human-adipose-tissue-derived mesenchymal stem cells (hAMSCs), preadipocytes, and adipocytes^4–6^. In addition, our recent investigation on the effect of UVA irradiation on hAMSCs has indicated that UVA irradiation can reduce the stemness of hAMSCs by downregulating the expression of stemness genes (OCT4, NANOG, and SOX2) through activating PGE_2_-cAMP-HIF-1α signaling^7^.

Two of the main characteristics of stem-cell biology are the self-renewal and differentiation potentials that are regulated by cell-cycling process involving numerous signaling molecules. Among these signaling molecules, it is well known that hypoxia-inducible factors (HIFs) contribute to self-renewal and differentiation processes of stem cells by regulating key transcription factors such as OCT4, NANOG, and SOX2^8, 9^. OCT4 is one of the PIT-OCT-UNC (POU)-family transcription factors originally defined on the basis of a common region of approximately 150 to 160 amino acids shared by mammalian transcription factors (PIT1, OCT1, and OCT2) and nematode transcription factor UNC86^10^. OCT4 regulates the differentiation of embryonic stem cells and maintains the pluripotent nature of blastocyst inner-cell mass. In addition, OCT4 can regulate genes that control stem-cell identity and differentiation by forming a complex with NANOG and SOX2^11^.

Macrophage migration inhibitory factor (MIF), one of the first pro-inflammatory cytokines identified, is expressed in various cell types and tissues^12, 13^. It is also induced by various noxious stimuli such as infection, inflammation, hypoxia, and UV irradiation^3, 14–16^. MIF is involved in the inhibition of macrophage. It also functions as a pleiotropic molecule^17^. Increasing evidence has indicated that MIF can regulate the proliferation and differentiation of several stem cells, including neural stem cells, cardiac stem cells, cartilage endplate stem cells, and adipos-derived stem cells^18–20^.

**Figure 1:**
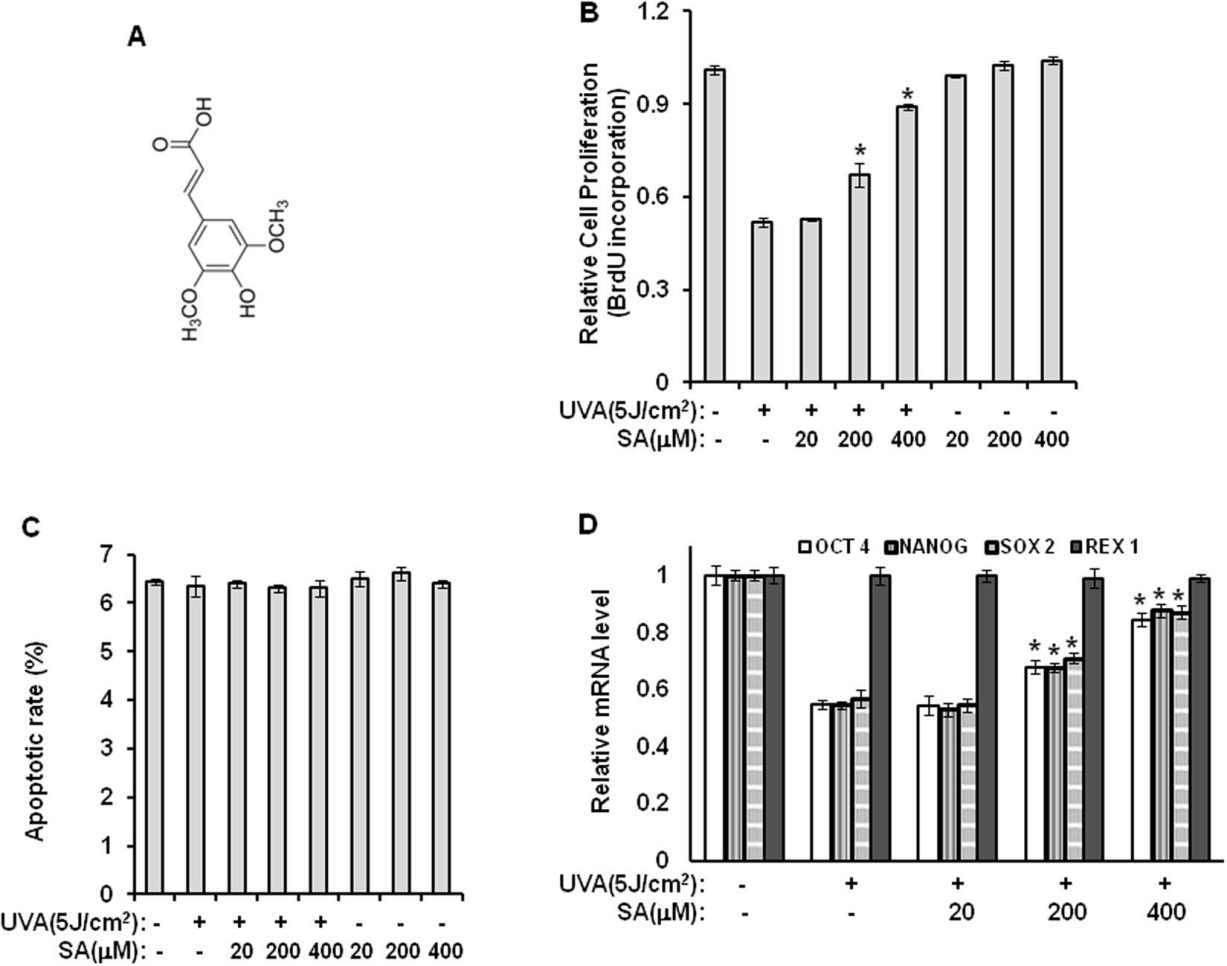
Sinapic acid reduces the effects of UVA irradiation on the proliferative potential and self-renewal of hAMSCs. hAMSCs were irradiated with 5 J/cm^2^ UVA and then incubated for three days in the presence of the indicated concentrations of sinapic acid under serum-free conditions. (**A**) Chemical structure of sinapic acid. (**B**) After three days, the cell proliferation was evaluated using the BrdU-incorporation assay. *p* < 0.05 vs. control. (**C**) The apoptotic effects of UVA irradiation were determined by Hoechst 33332 staining. *p < 0.05 vs. control. (**D**) After three days of incubation under serum-free conditions, the total RNA was isolated, and the mRNA levels of the indicated genes were measured using a real-time quantitative RT-PCR. The expressed results are relative to the untreated cells after the normalization against the GAPDH level. *p < 0.05 vs. UVA (5 J/cm^2^)-treated control. The results were confirmed by four independent experiments. Each experiment was conducted in duplicate. Data are expressed as mean ± S.D. SA: sinapic acid.

As an approach to attenuate harmful effects of UVA irradiation on stemness, hypoxia responsive element (HRE)-luciferase reporter assay was conducted to screen and evaluate tested materials for their UVA-antagonist effects. During this screening, sinapic acid (3,5-dimethoxy-4-hydroxycinnamic acid) was selected as a potential UVA-antagonist candidate (Fig. 1A). Sinapic acid is a naturally occurring member of the phenylpropanoid family. It is found in a variety of edible plants such as spices, berry fruits, citruses, vegetables, cereals, and oilseed crops^21–25^. It has been reported that sinapic acid possesses several beneficial properties against various pathological conditions including inflammation, infections, diabetes, oxidative stress, cancer, anxiety, and neurodegeneration^26–33^. However, the effect of sinapic acid on stemness and differentiation potential of stem cells has not been reported yet.

Therefore, the aim of this study was to explore the antagonist effect of sinapic acid on UVA-irradiation-induced damage to stemness and differentiation potential of hAMSCs and investigate its UVA-antagonist mechanisms.

## Results

### Sinapic acid antagonizes the effects of UVA irradiation on cell proliferation and self-renewal potential of hAMSCs

In this study, cell-proliferation assay and real-time PCR analysis were performed to investigate the effect of sinapic acid on UVA-induced reduction in stemness of hAMSCs. As shown in Fig. 1B, UVA-induced reduction of cell proliferation was recovered by sinapic-acid treatment in a concentration-dependent manner. However, sinapic acid showed no apoptotic effects on cells at tested concentrations (Fig. 1C). In addition, the reduced expression levels of OCT4, NANOG, and SOX2 induced by UVA irradiation were all increased by sinapic-acid treatment (Fig. 1D), suggesting that sinapic acid could antagonize the damaging effects of UVA irradiation on hAMSCs.

### Sinapic acid restores the expression of HIF-1α downregulated by UVA irradiation

**Figure 2:**
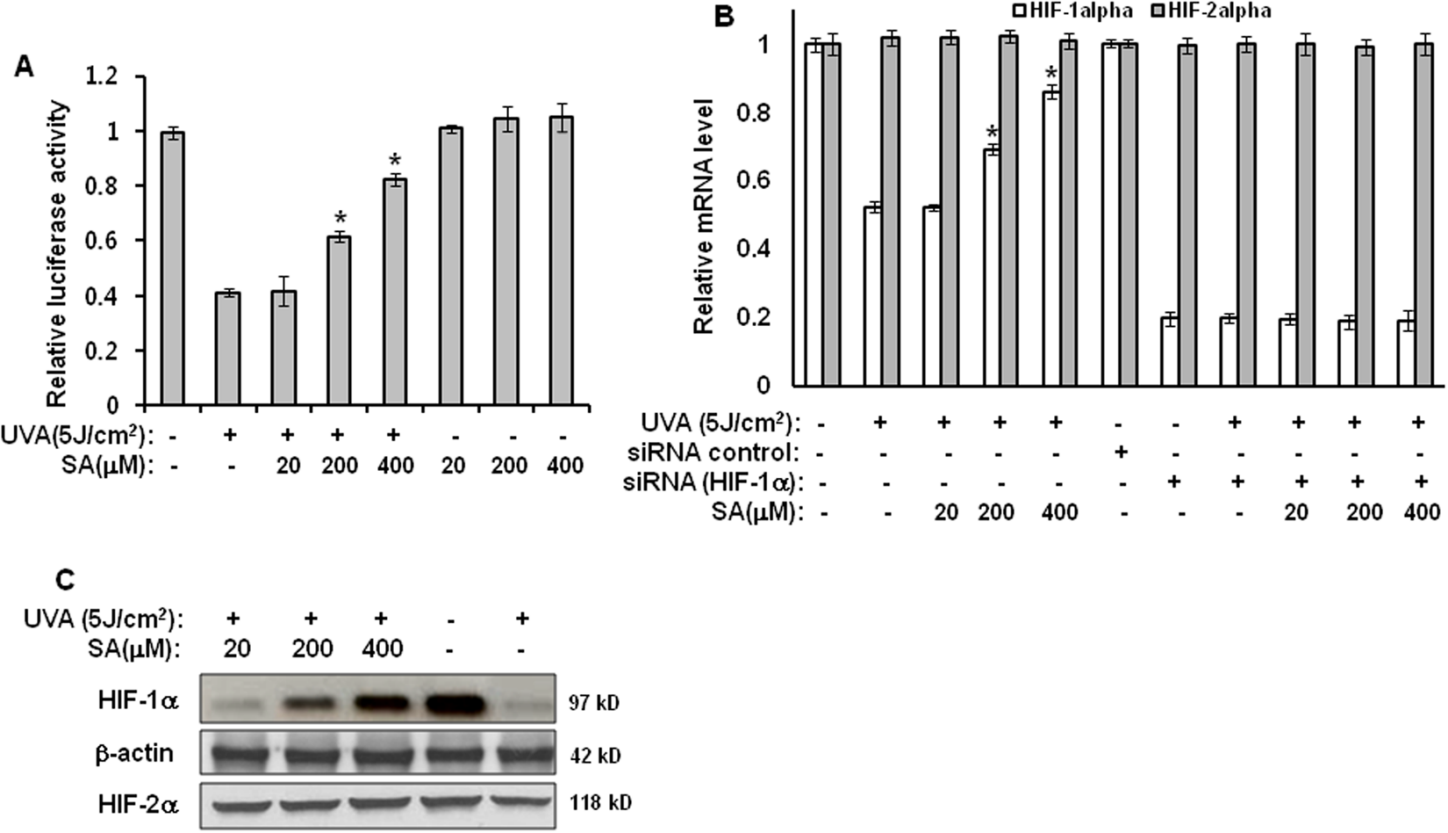
Sinapic acid increases the downregulated expression of HIF-1α that is caused by UVA irradiation. (**A**) hAMSCs were transfected with the HRE-Luc reporter along with a Renilla-luciferase expression vector that was driven by a thymidine-kinase promoter using the DharmaFECT^®^ Duo transfection reagent according to the manufacturer’s protocols. After an incubation time of 24 h, the cells were irradiated with 5 J/cm^2^ UVA and then further incubated in the presence of the indicated concentrations of sinapic acid under serum-free conditions for 14 h. These cells were then harvested, lysed, and assayed. *p < 0.05 vs. UVA (5 J)-treated control. (**B**) hAMSCs were irradiated with 5 J/cm^2^ UVA, or they were transfected with the siRNA for HIF-1α or HIF-2α, and then incubated for three days in the presence of the indicated concentrations of sinapic acid under serum-free conditions. After three days of incubation, the mRNA levels of the HIF-1α and HIF-2α genes were measured using a real-time quantitative RT-PCR. The expressed results are relative to the untreated cells after the normalization against the GAPDH level. *p < 0.05 vs. UVA (5 J/cm^2^)-treated control. (**C**) The total lysates were analyzed using the western blot for which the anti-HIF-1α and anti-HIF-2α antibodies were used. All of the results were verified by repeating the experiments three times. Data are expressed as mean ± S.D. SA: sinapic acid.

To investigate the effect of sinapic acid on the expression of HIF-1α reduced by UVA irradiation, HRE-luciferase reporter assay, real-time PCR, and western-blot analyses were performed to determine the expression of HIF-1α and HIF-2α genes. Luciferase-reporter assay results showed that the activity of HRE-luciferase reporter reduced by UVA irradiation was increased by treatment with sinapic acid (Fig. 2A). In addition, treatment with sinapic acid attenuated the reduction of mRNA and protein levels of HIF-1α induced by UVA irradiation (Fig. 2B,C). However, treatment with sinapic acid did not affect the expression levels of HIF-2α (Fig. 2B,C). Taken together, these results suggest that sinapic acid could recover stemness of hAMSCs reduced by UVA irradiation through upregulation of HIF-1α.

### Sinapic acid reduces UVA-induced production of PGE_2_ and cAMP through inhibition of p38 MAPK and NF-κB

**Figure 3:**
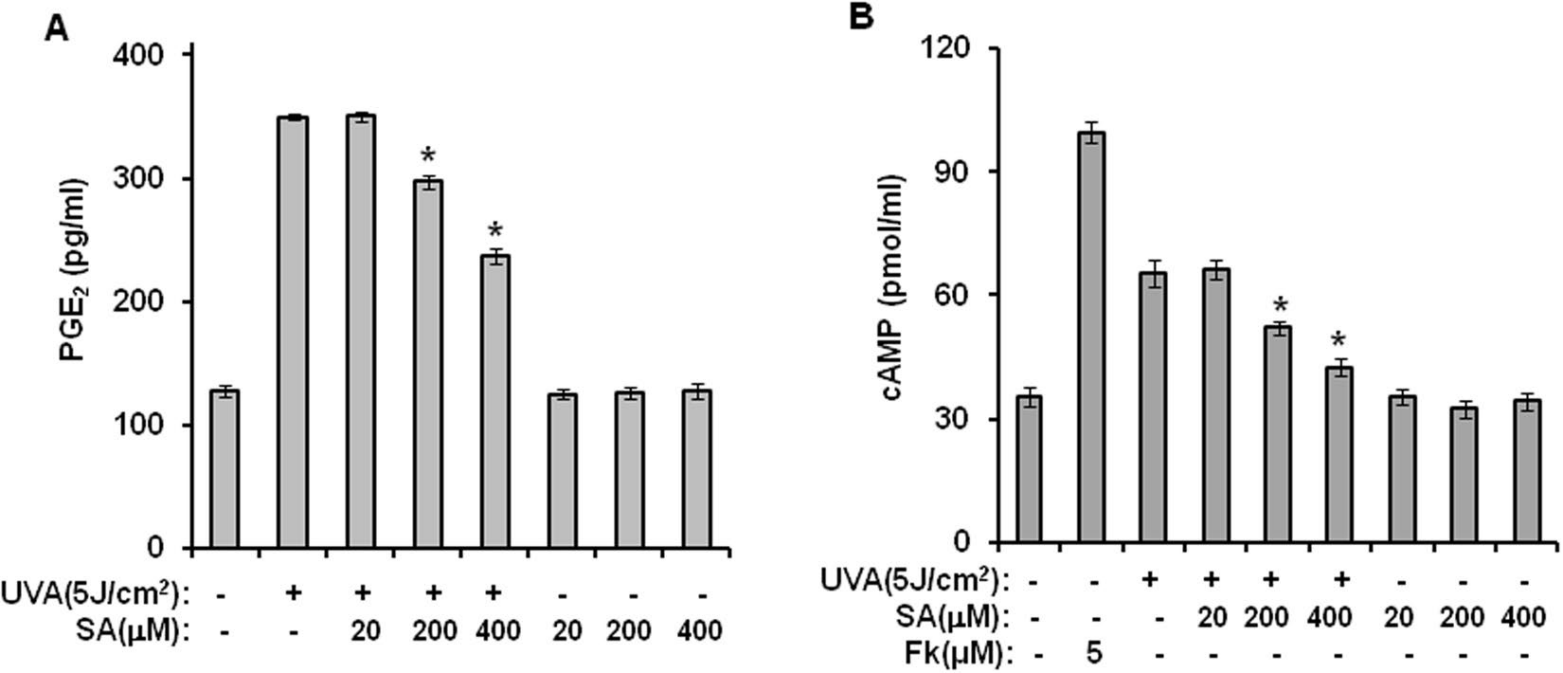
Sinapic acid reduces the UVA-induced production of PGE_2_ and cAMP. hAMSCs were irradiated with 5 J/cm^2^ UVA and then incubated for three days in the presence of the indicated concentrations of sinapic acid under serum-free conditions. (**A**,**B**) After three days of incubation, the supernatants were harvested for the PGE_2_ (**A**) and cAMP (**B**) measurements. Data are expressed as mean ± S.D. *p < 0.05 vs. UVA (5J)-treated control. The results were verified by repeating the experiments, each of which was conducted in duplicate, three times. SA: sinapic acid.

**Figure 4:**
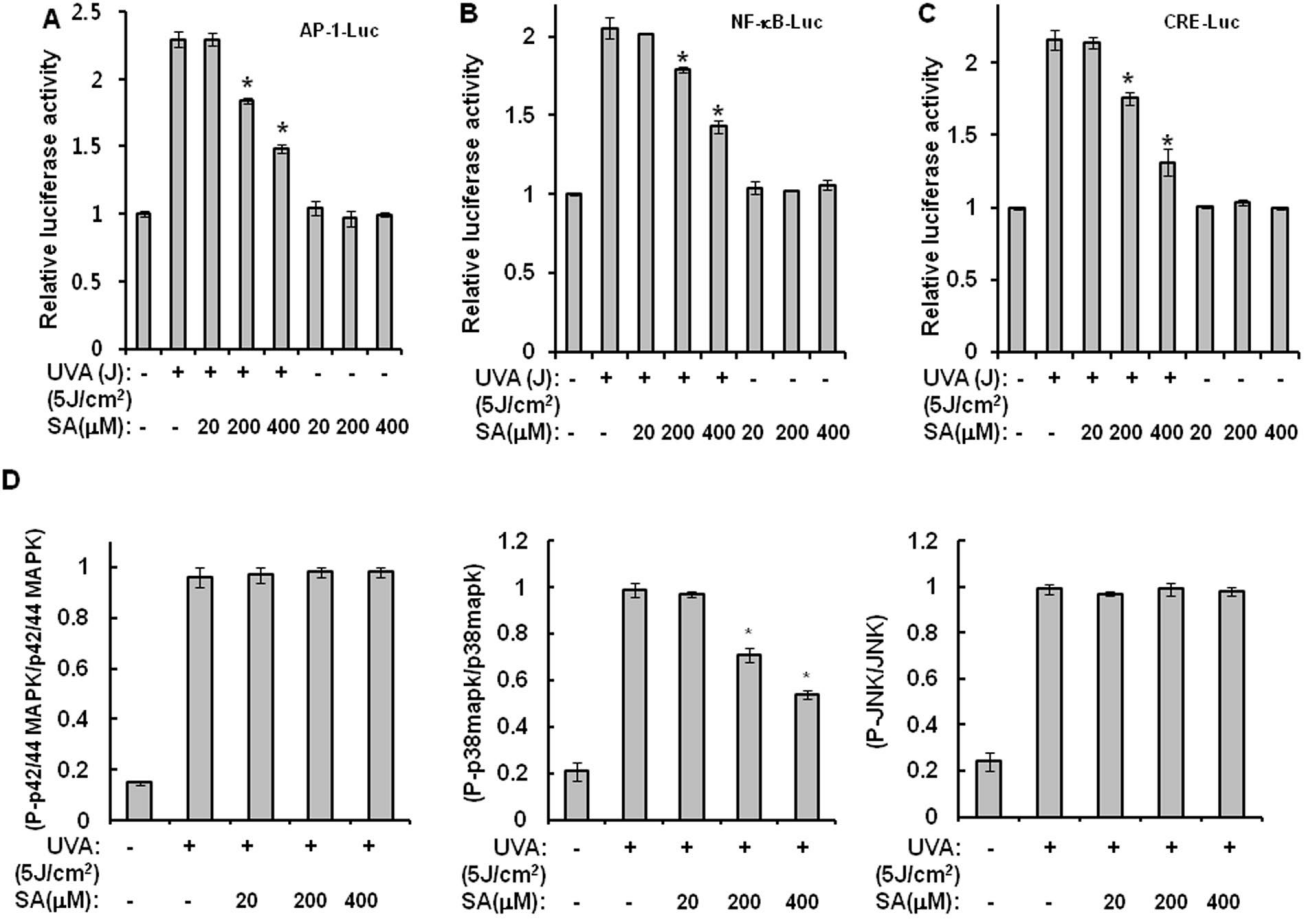
Sinapic acid reduces the UVA-induced production of PGE_2_ and cAMP through inhibition of NF-κB and p38 MAPK. (**A**–**C**) hAMSCs were transfected with AP-Luc, CRE-Luc, or NF-κB-Luc reporters along with a Renilla-luciferase expression vector driven by a thymidine-kinase promoter for which the DharmaFECT^®^ Duo transfection reagent was used according to the manufacturer’s protocols. After an incubation time of 24 h, the cells were irradiated with 5 J/cm^2^ UVA and then further incubated in the presence of the indicated concentrations of sinapic acid under serum-free conditions for 14 h. These cells were then harvested, lysed, and assayed. *p < 0.05 vs. UVA (5 J/cm^2^)-treated control. (**D**) hAMSCs were irradiated with 5 J/cm^2^ UVA and then treated with the indicated concentrations of sinapic acid for 1 h under serum-free conditions. After an incubation time of 1 h, the cell lysates were analyzed using the Multi-Target Sandwich ELISA Kit. All of the results were verified by repeating the experiments, each of which was conducted in duplicate, three times. SA: sinapic acid.

The results in the previous section revealed that sinapic acid could restore UVA-irradiation-induced reduction of HIF-1α. PGE_2_ and cAMP are well-known upstream molecules of HIF-1α. Therefore, the effect of sinapic acid on UVA-irradiation-induced production of PGE_2_ and cAMP was examined. As shown in Fig. 3A,B, treatment of hAMSCs with sinapic acid significantly reduced production levels of PGE_2_ and cAMP when compared to UVA-irradiated controls without treatment with sinapic acid. Luciferase-reporter assays were conducted for AP-1, CRE, and NF-κB to determine the action mechanisms of sinapic acid. As shown in Fig. 4, sinapic acid reduced the activations of AP-1, CRE, and NF-κB caused by UVA irradiation, suggesting that sinapic acid could attenuate UVA-induced production of PGE_2_ through inhibiting activities of AP-1 and NF-κB. In addition, among three types of MAPKs evaluated, p38 MAPK, but not JNK or p42/44 MAPK, showed reduced activity after treatment with sinapic acid when compared to that in UVA-irradiated controls (Fig. 4D). Collectively, these results suggest that sinapic acid could attenuate UVA-induced reduction in stemness by reducing production levels of PGE_2_ and cAMP via inhibiting NF-κB and p38 MAPK.

### Antagonist effect of sinapic acid against UVA-induced downregulation of stemness genes by downregulating PGE_2_-cAMP-HIF-1α signaling through inhibition of AP-1 and NF-κB

**Figure 5:**
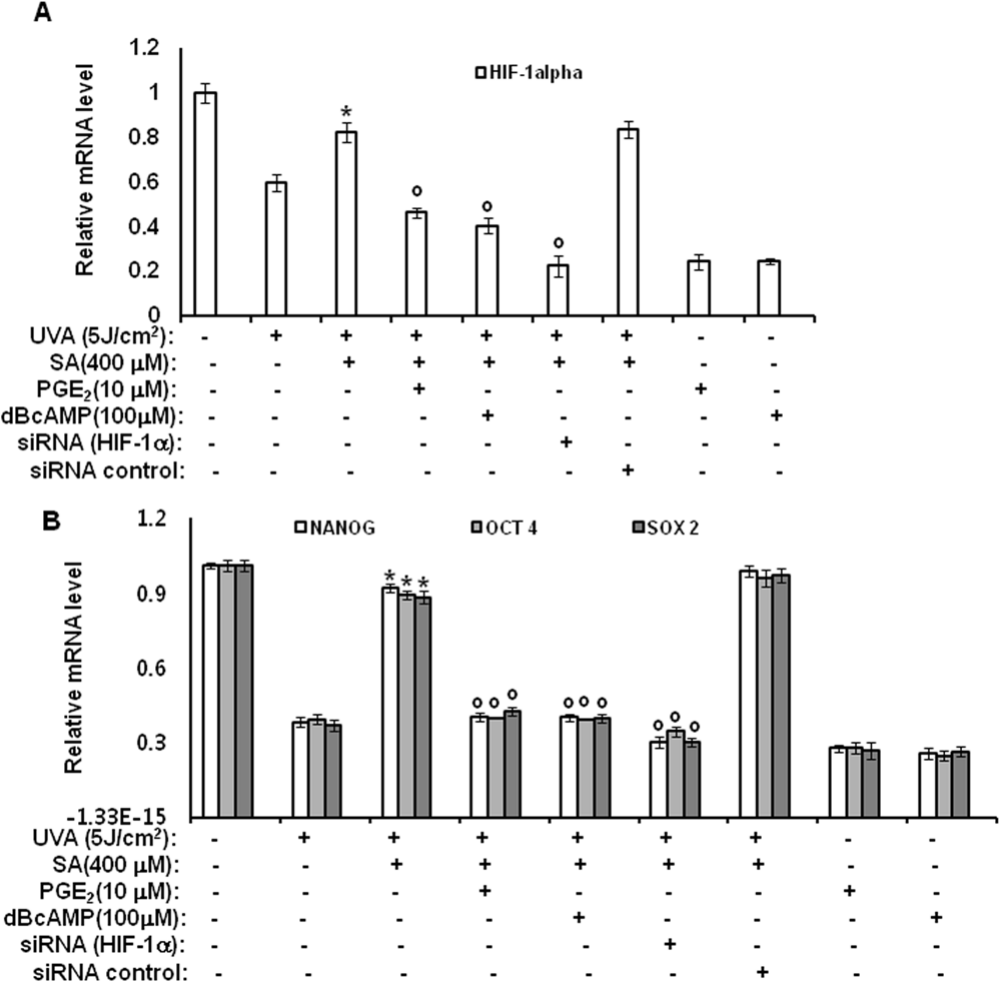
Sinapic-acid effects against the UVA-induced downregulation of the stemness genes are mediated by downregulation of PGE_2_-cAMP-HIF-1α signaling through inhibition of AP-1 and NF-κB. hAMSCs were irradiated with 5 J/cm^2^ UVA, or transfected with the siRNA for HIF-1α, and then incubated for three days with sinapic acid (100 μM) in the presence of the indicated concentration of PGE_2_ or cAMP under serum-free conditions. The mRNA levels of the HIF-1α gene (**A**) and the OCT4, NANOG, and SOX2 genes (**B**) were measured using a real-time quantitative RT-PCR. The expressed results are relative to the untreated cells after the normalization against the GAPDH level. Data are expressed as mean ± S.D. *p < 0.05 vs. UVA (5 J/cm^2^)-treated control. °*p* < 0.05 vs. UVA (5 J/cm^2^)- and sinapic acid (100 μM)-treated controls. The results were verified by repeating the experiments, each of which was conducted in duplicate, four times. SA: sinapic acid; dBcAMP: dibutyryl cAMP.

**Figure 6:**
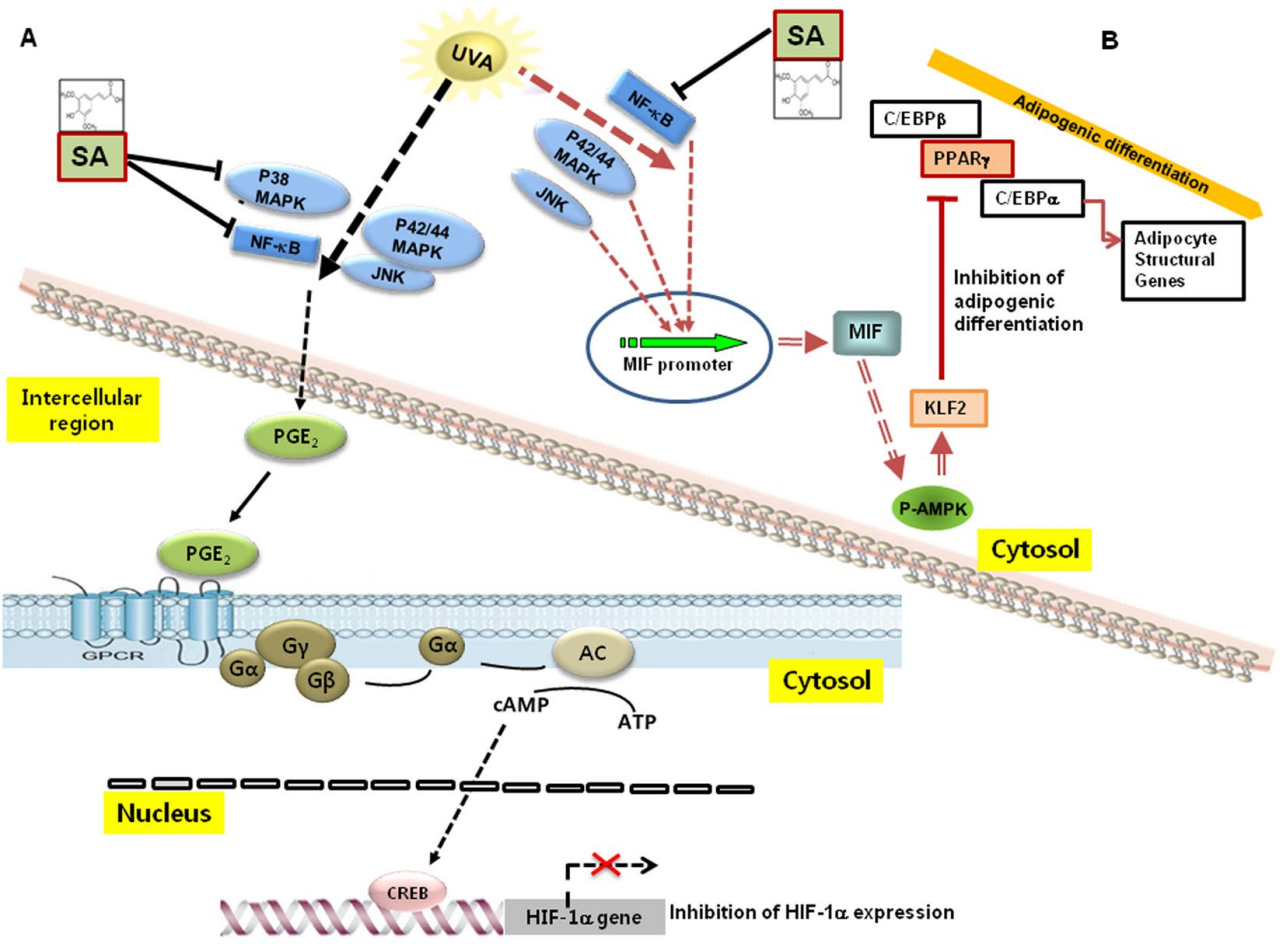
Mechanisms involved in the antagonist effect of sinapic acid against UVA-induced attenuation of stemness and differentiation properties of stem cells. (**A**) UVA irradiation induces the production of PGE_2_ and its downstream molecule, cAMP, through the activations of AP-1 and NF-κB. The cAMP molecule sequentially reduces the expression of the HIF-1α gene through the CREB activation that consequently downregulates the expression of the stemness genes such as NANOG, SOX2, and OCT4. In the UVA-irradiation-induced signaling pathway, sinapic acid attenuates the UVA-induced effects on the expression of the stemness genes by inhibiting p38 MAPK and NF-κB, which is upstream of the PGE_2_ production. AC: adenylate cyclase. (**B**) Under adipogenic condition, UVA irradiation induces the expression of MIF through the activations of p42/44 MAPK, JNK, and NF-κB. The MIF molecule then induces the phosphorylation of the AMPK protein, consequently upregulating the expression of KLF gene. The KLF protein downregulates the expression of PPARγ, an adipogenic factor. In the UVA-irradiation-induced signaling pathway under adipogenic condition, sinapic acid attenuates the UVA-induced inhibition of adipogenic differentiation of stem cells by inhibiting the production of MIF through the suppression of NF-κB.SA: sinapic acid, UVA: ultraviolet.

The previous section revealed that sinapic acid could enhance the expression levels of stemness genes by downregulating PGE_2_-cAMP-HIF-1α signaling through inhibition of NF-κB and p38 MAPK. To further determine the roles of HIF-1α in the effect of sinapic acid on stemness, RNA-interference experiments were performed using specific siRNAs for HIF-1α. As shown in Fig. 5, treatment with sinapic acid reduced the effects of UVA irradiation regarding the expression levels of the stemness-related genes. Specifically, the expression levels of HIF-1α gene (Fig. 5A) and stemness-related genes (OCT4, NANOG, and SOX2) (Fig. 5B) reduced by UVA irradiation were increased by treatment with sinapic acid. However, such effects of sinapic acid were attenuated by treatments with PGE_2_, and cAMP as well as knock-down of HIF-1α gene by siRNA. These results indicate that sinapic acid operates upstream of PGE_2_, cAMP, and HIF-1α molecules, suggesting that the effects of sinapic acid are mediated by downregulation of PGE_2_-cAMP-HIF-1α signaling through inhibition of AP-1 and NF-κB. The possible action mechanisms of sinapic acid involved in its antagonistic effect on UVA-irradiation are shown in Fig. 6A.

### Sinapic acid reduces UVA-induced suppression of adipogenic differentiation through downregulating MIF-AMPK-KLF2 signaling

**Figure 7:**
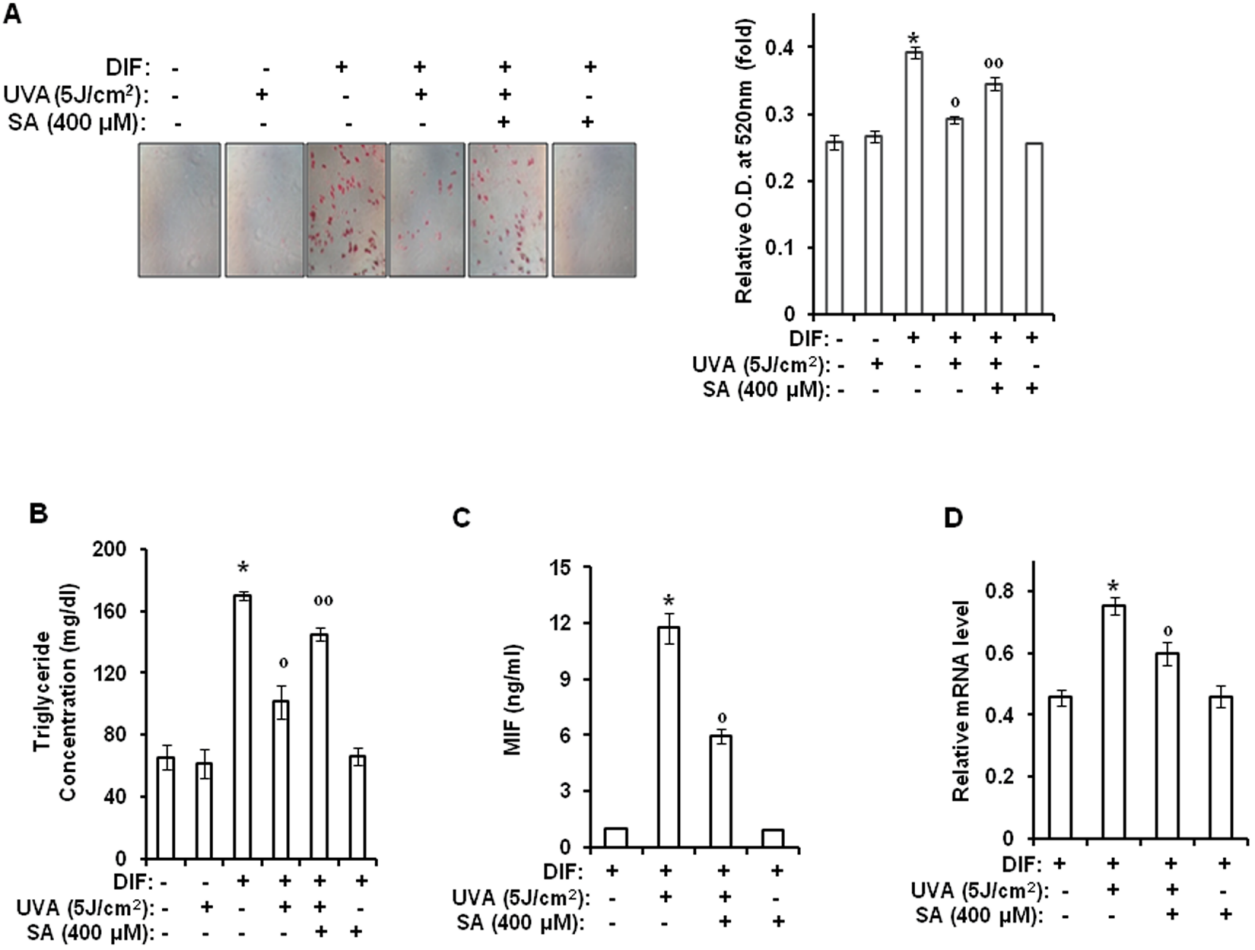
Sinapic acid attenuates UVA-induced suppression of adipogenic differentiation through downregulation of MIF gene expression. Two-day post confluent hAMSCs (day 0) were irradiated with UVA (5 J/cm^2^) and then treated with sinapic acid (400 μM), followed by stimulation with STEM PRO^®^ adipocyte differentiation media for 3 days. The medium was then replaced with STEM PRO^®^ adipocyte differentiation media every three days until the end of the experiment at day 14. These assays were performed on fully differentiated adipocytes (day 14). (**A**) Intracellular lipids were stained with Oil Red O. The results were confirmed by three independent experiments, which were each conducted in duplicate. (**B**) The triglyceride content was measured using a triglyceride assay kit (Cayman Chemical, Ann Arbor, MI). The results were verified by three repetitions of the experiments, each of which was conducted in triplicate. (**C**) At day 14 after the induction of differentiation, the supernatants were harvested for MIF measurement. Data are expressed as the means ± S.D. (**D**) At 14 days after the induction of differentiation, the total RNA was isolated and the mRNA levels of the MIF gene were measured by real-time quantitative RT-PCR. The results were verified by four repetitions of the experiments, each of which was conducted in triplicate. *p < 0.05 vs. DIF-untreated control. ^°^ *p* < 0.05 vs. DIF-treated control. ^°°^ *p* < 0.05 vs. UVA (5 J/cm^2^)-treated control. The results were verified by three repetitions of the experiments, each of which was conducted in duplicate. SA: sinapic acid, DIF: differentiation media, UVA: ultraviolet A.

Results of the present study indicated that UVA irradiation could reduce stemness by activating PGE_2_-cAMP signaling and such effect could be attenuated by sinapic acid by downregulating PGE_2_-cAMP signaling. It has been reported that cAMP signaling can promote the differentiation of stem cells^34, 35^, suggesting that UVA irradiation might be involved in the differentiation process. However, as shown in Fig. 7A,B, UVA irradiation failed to promote adipogenic differentiation of hAMSCs. On the contrary, UVA irradiation inhibited adipogenic differentiation. Specifically, Oil Red O staining revealed that lipid accumulation in UVA-irradiated cells was significantly lower than that in control cells without UVA-irradiation (Fig. 7A). Results of cellular triglyceride content assay revealed that UVA irradiation inhibited triglyceride accumulation on day 21 after full differentiation occurred (P < 0.05) (Fig. 7B). These results indicated that UVA irradiation could inhibit adipogenic differentiation of stem cells, consistent with our previous report demonstrating that UVA irradiation had anti-adipogenic effects^3^. However, treatment with sinapic acid reduced the anti-adipogenic effects of UVA, thus having differentiation potential-protection effect (Fig. 7A,B).

**Figure 8:**
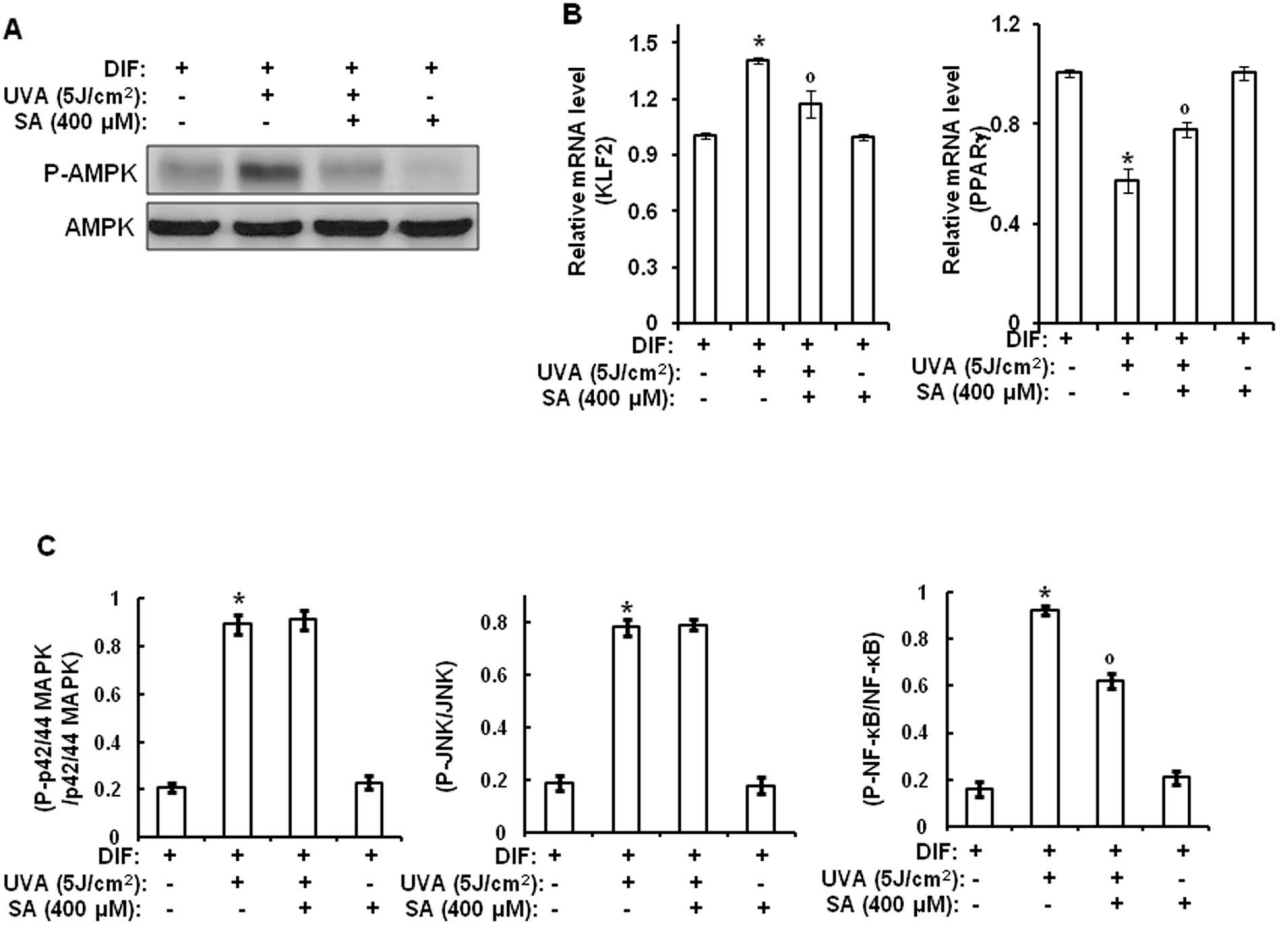
Sinapic acid attenuates UVA-induced suppression of adipogenic differentiation through downregulation of MIF-AMPK-KLF2 signaling by inhibiting NF-κB. Two-day post confluent hAMSCs (day 0) were irradiated with UVA (5 J/cm^2^) and then treated with sinapic acid (400 μM), followed by stimulation with STEM PRO^®^ adipocyte differentiation media for 3 days. The medium was then replaced with STEM PRO^®^ adipocyte differentiation media every three days until the end of the experiment at day 14. These assays were performed on fully differentiated adipocytes (day 14). (**A**) At 14 days after the induction of differentiation, the total lysates were analyzed by Western blot using the indicated antibodies. (**B**) At 14 days after the induction of differentiation, the total RNA was isolated and the mRNA levels of the KLF2 and PPARγ genes were measured by real-time quantitative RT-PCR. The results were verified by four repetitions of the experiments, each of which was conducted in triplicate. (**C**) At 1 h after the induction of differentiation, cell lysates were analyzed using a Multi-Target Sandwich ELISA Kit. All of the results were verified by repeating the experiments, each of which was conducted in duplicate, three times. SA: sinapic acid, DIF: differentiation media, UVA: ultraviolet A. Data are expressed as the means ± S.D. *p < 0.05 vs. DIF-untreated control. ^°^ *p* < 0.05 vs. DIF-treated control. ^°°^ *p* < 0.05 vs. UVA (5 J/cm^2^)-treated control.

Our group has previously reported that UVA irradiation can activate MIF-AMPK-KLF2 signaling, thus inhibiting the adipogenic differentiation of hAMSCs^3^. Using this system, we examined the effect of sinapic acid on UVA-induced MIF-AMPK-KLF2 signaling. As shown in Fig. 7C,D, UVA irradiation increased both mRNA and protein levels of MIF. However, sinapic acid reduced such effects of UVA irradiation. UVA-induced phosphorylation of AMPK, a downstream molecule of MIF, was also suppressed by sinapic acid treatment (Fig. 8A). We also examined the effect of sinapic acid on mRNA levels of KLF2, an anti-adipogenic regulator, and PPARγ, an adipogenic factor. As shown in Fig. 8B, UVA irradiation increased the mRNA levels of KLF2 but decreased the mRNA levels of PPARγ. However, sinapic acid reduced such effects of UVA on the expression level of KLF2 and PPARγ. These results suggest that sinapic acid could protect the differentiation potential of stem cells against UVA irradiation.

### Sinapic acid reduces UVA-induced MIF-AMPK-KLF2 signaling through inhibition of NF-κB but not MAPKs

Results of the present study indicated that sinapic acid could reduce UVA-induced suppression of adipogenic differentiation through downregulating MIF-AMPK-KLF2 signaling. UVA irradiation has been reported to be able to activate p42/44 MAPK, JNK, and NF-κB during adipogenic differentiation^3^. Therefore, we investigated the involvement of these molecules in the antagonizing effects of sinapic acid against UVA irradiation. As shown in Fig. 6H, UVA irradiation activated p42/44 MAPK, JNK and NF-κB during adipogenic differentiation. In addition, the activity of NF-κB, but not JNK or p42/44 MAPK, was reduced by sinapic acid when compared to that in UVA-irradiated controls (Fig. 8C). Collectively, these results suggest that sinapic acid could attenuate UVA-induced reduction in differentiation potential by reducing MIF-AMPK-KLF2 signaling via inhibition of NF-κB. The possible action mechanisms involved in the antagonizing effect of sinapic acid on UVA-irradiation are shown in Fig. 6B.

## Discussion

It has been reported that UVA-irradiation can suppress the stemness properties of stem cells^7^. The action mechanisms involve an increase of PGE_2_ production via activation of AP-1 and NF-κB^7^. Specifically, reduced stemness has been associated with reduced expression of HIF-1α gene caused by UVA irradiation. In this study, we demonstrated that sinapic-acid treatment could attenuate these UVA-irradiation effects by reducing the production of PGE_2_ through inhibiting NF-κB and p38 MAPK, thus increasing the stemness properties of hAMSCs. In addition, sinapic acid suppressed the inhibition of adipogenic differentiation induced by UVA irradiation by reducing the expression levels of MIF via inhibiting NF-κB. Collectively, the inhibitory effects of sinapic acid on NF-κB and p38 MAPK contributed to the recovery of stemness and differentiation potential of stem cells damaged by UVA irradiation.

The expression of HIFs plays an important role in the regulation of stemness properties of stem cells. Among several pathways involved in stemness, the PGE_2_-cAMP signaling pathway can downregulate the expression of HIFs and inhibit stemness^36, 37^. In addition, UVA irradiation can attenuate stemness by downregulating the expression of HIF-1α through activating the PGE_2_-cAMP signaling pathway, which is dependent on AP-1 and NF-κB^7^. This study was conducted to examine the effects of sinapic acid on UVA-induced PGE_2_-cAMP signaling pathway. The results showed that sinapic acid could suppress UVA-induced productions of PGE_2_ and cAMP, a downstream molecule of PGE_2_, by inhibiting NF-κB and p38 MAPK. These results indicate that sinapic acid could attenuate the damaging effects of UNA by selectively inhibiting the activation of NF-κB and p38 MAPK. Therefore, sinapic acid might have potential as a UVA-antagonist agent to recover the stemness of hAMSCs.

NANOG, OCT4, and SOX2 are important transcription factors that regulate stem-cell self-renewal^38^. Therefore. they are also called “stemness genes”. Their expression levels are regulated by HIFs whose expression is downregulated by UVA irradiation^7^. In this study, the expression levels of OCT4, NANOG, and SOX2 were reduced by UVA irradiation. However, their expression levels were increased by treatment with sinapic acid. These results suggest that sinapic acid could attenuate the damaging effects of UVA irradiation on hAMSCs by increasing the expression levels of OCT4, NANOG, and SOX2 genes.

The expression of MIF is involved in the regulation of differentiation properties of stem cells. Among several pathways involved in differentiation, the MIF-AMPK signaling pathway downregulates the differentiation of stem cells^19, 39^. In addition, UVA irradiation attenuates the differentiation potential by increasing MIF expression through activating the AMPK-KLF2 signaling pathway which is dependent on p42/44 MAPK, JNK, and NF-κB^3^. This study also investigated the effects of sinapic acid on UVA-induced MIF-AMPK-KLF2 signaling pathway. The results showed that sinapic acid could suppress expression of MIF and AMPK phosphorylation induced by UVA irradiation. In addition, UVA-induced expression of KLF2, a downstream molecule of AMPK, was suppressed by sinapic acid. These results indicate that sinapic acid could attenuate the effects of UVA under adipogenic condition by selectively inhibiting the activation of NF-κB. Therefore, sinapic acid may have potential as a UVA-antagonist agent to recover the differentiation potential of hAMSCs.

It has been reported that cAMP signaling can promote the differentiation of stem cells^34, 35^. Our data also indicated that UVA irradiation induced the activation of PGE_2_-cAMP signaling. Therefore, UVA irradiation might be able to induce the differentiation process of stem cells. However, our results showed that UVA irradiation failed to induce the differentiation of hAMSCs. This might be attributed to different conditions. Under serum-free condition, UVA irradiation induced cAMP signaling. However, cAMP signaling was not significantly activated by UVA irradiation under adipogenic condition.

UVA irradiation exerts various effects on cells and tissues^1–3^. In particular, it has been reported that UVA irradiation inhibits adipogenic differentiation^3^ and the stemness of hAMSCs^7^, suggesting that UVA irradiation is deleterious to normal stemness biology of hAMSCs. Recently, stem cells have received a large amount of attention as a therapeutic candidate for cell therapy. The stem-cell industry is rapidly growing. For this reason, it is very important to culture stem cells in large quantities and maintain their stemness irrespective of external stresses such as those from UVA and oxidation. Several studies have reported that natural compounds such as vanillin and aspartic acid could protect stem cells against UVA irradiation^40, 41^. Results of this study showed that sinapic acid could attenuate the damaging effect of UVA irradiation on stemness by selectively inhibiting the activation of NF-κB and p38 MAPK. It also could attenuate the damaging effect of UVA irradiation on differentiation potential of stem cells by suppressing the activation of NF-κB. Therefore, sinapic acid might be useful as an agent to restore UVA-damaged stemness and differentiation properties of hAMSCs.

In conclusion, results of this study indicate that sinapic acid could attenuate the damaging effects of UVA irradiation by reducing the production of PGE_2_ through inhibiting NF-κB and p38 MAPK and by reducing the production of MIF through suppression of NF-κB, thereby recovering the stemness and differentiation properties of hAMSCs.

## Materials and Methods

### Human-adipose-tissue-derived stem-cell culture

Three kinds of hAMSCs were purchased from Invitrogen (Carlsbad, CA, USA), ATCC (Manassas, VA, USA), and Thermo Fisher Scientific, Inc. (Waltham, MA, USA). These cryopreserved cells were thawed at 37 °C and immediately cultured in MesenPRO RS^TM^ medium (Gibco, Carlsbad, CA, USA). These cells were then expanded using the MesenPRO RS^TM^ medium through five passages. The medium was changed every three days until cells were 70% confluent, at which time they were passaged.

### Adipogenic stimulation of hAMSCs

hAMSCs were seeded into 6 cm diameter cell culture dishes at a density of 15 × 10^4^ cells/well. Cells were grown in MesenPro RS^TM^ media (Invitrogen) at 37 °C under 5% CO_2_. To induce differentiation, 2-day post confluent hAMSCs (day 0) were incubated for 15 days with STEM PRO adipocyte differentiation media (Invitrogen). To examine the effect of sinapic acid on adipocyte differentiation of hAMSCs, 2-day post confluent hAMSCs were treated with sinapic acid at indicated concentrations and then stimulated with STEM PRO adipocyte differentiation media for 3 days. The medium was replaced with STEM PRO adipocyte differentiation media every 3 days until the end of the experiment on day 14.

### Oil red O staining

hAMSCs that had been treated as described above were washed with PBS and then fixed with 10% formalin for 30 min. These cells were then washed twice with distilled water and stained for at least 1 h at room temperature in freshly diluted Oil Red O solution (six parts Oil Red O stock solution and four parts H_2_O; Oil Red O stock solution is 0.5% Oil Red O in isopropanol). Stained cells were air dried overnight and then dissolved in 1-butanol for OD detection at wavelength of 520 nm.

### Triglyceride assay

hAMSCs that had been treated as described above were washed with PBS and harvested in 25 mM Tris buffer (pH 7.5) containing 1 mM EDTA. Samples were then sonicated three times for 15 seconds each using a UP50H instrument with a MS7 (Hielscher Ultrasonics GmbH, Teltow, Germany) to homogenize cell suspension, after which total triglyceride content was evaluated using a triglyceride assay kit (Cayman Chemical, Ann Arbor, MI, USA). Protein content in an aliquot of the homogenate was also determined using a protein assay kit (Pierce, Rockford, IL, USA).

### UVA irradiation

For UVA exposure, the medium was removed when cells were 70% confluent. Cells were then washed with PBS and gently overlaid with Dulbecco’s modified Eagle’s medium (DMEM) devoid of phenol red (Santa Cruz Biotechnology, Inc., Santa Cruz, CA, USA). These cells were then irradiated from 0.05 J/cm^2^ to 5 J/cm^2^ (not cytotoxic) for 21 sec to 36 min. The UVA irradiation was conducted in the DMEM devoid of phenol red using a Vilber-Lourmat UVA table centered on 365 nm (TF-20L) at 25 °C that was controlled during the irradiation. During the irradiation, the lid of the dish was open to minimize the UVA absorption by the plastic materials. A piece of glass of a 4 mm thickness was placed above the table to absorb the residual UVB radiation.

### Cell proliferation

The hAMSCs were irradiated with the indicated UVA doses before they were incubated with 20 μM to 400 μM of sinapic acid (purity: 98%, Sigma–Aldrich, St. Louis, MO, USA) for three days under serum-free conditions (in the DMEM devoid of serum, at 37 °C with 5% CO_2_). The serum-free conditions were chosen to exclude the unknown effects of the exogenous serum that might have various compositions depending on the donor species, the age of the animal the serum was obtained from and its feedstock, and the season. After three days, the cell proliferation was measured using the BrdU-incorporation assay. The ELISA-based detection of the BrdU incorporation was performed using the BrdU Cell Proliferation Assay Kit (Cell Signaling Technology, Danvers, MA, USA) according to the manufacturer’s instructions.

### Assessment of the percentages of the apoptotic cells and necrotic cells

To detect the apoptotic and necrotic cells, the Apoptotic/Necrotic/Healthy Cells Detection Kit was used (PromoCell GmbH, Heidelberg, Germany). The cells were stained with Hoechst 33342, FITC-Annexin V, and Ethidium Homodimer. This assay was conducted after the hAMSCs were incubated under the conditions described above. After washing with a 1X binding buffer, the cells were observed under a fluorescence microscope (Zeiss Axiophoto 2, Carl Zeiss, Germany). In this assay, the apoptotic cells were stained both green and blue, while the necrotic cells were stained both red and blue. A minimum of 500 cells was scored from each sample.

### Small-interference RNA (siRNA) and the expression plasmid for HIF-1α

The ON-TARGETplus SMARTpool human siRNAs against HIF-1α (L-004018-00-0020), HIF-2α (L-004814-00-0020), and ON-TARGETplus non-targeting siRNA (D-001810-10-05) were synthesized by Thermo Fisher Scientific, Inc. (Waltham, MA, USA). The expression plasmid for HIF-1α was purchased from OriGene (Rockville, MD, USA). The cells were then transfected with the indicated siRNAs at 50 nM, or along with the HIF-1α-expression plasmid for 48 h using the DharmaFECT transfection agent (Dharmacon Research, CO, USA), according to the manufacturer’s instructions.

### RNA preparation and cDNA synthesis

The total cellular RNA was extracted from the hAMSCs that had been grown in the serum-free culture medium, with or without the indicated concentrations ofsinapic acid, and in the presence or absence of the indicated UVA-irradiation doses, for three days using the TRIzol reagent (Invitrogen, Carlsbad, CA, USA), followed by a purification using the RNeasy Mini Kit (Qiagen, CA, USA) according to the manufacturer’s instructions. All of the samples were DNase-treated (Ambion, CA, USA), followed by subsequent analyses on the Agilent Bioanalyzer (Agilent Technologies, Waldbronn, Germany) and the NanoDrop 8000 Spectrophotometer (Thermo Scientific, Schwerte, Germany) to determine the RNA concentration, purity, and integrity. The samples with the appropriate RNA-integrity number (RIN, ≧8.0) and RNA purity (A_260_/A_280_ = 1.8 to 2.0) were used. The purified RNA (1 μg) was reverse-transcribed in a 20 μl reaction mixture using the RevertAid^TM^ First Strand cDNA Synthesis Kit Oligo dT Primers (Fermentas, Canada) on a BioRad PTC-200 DNA Engine thermal cycler (BioRad, Hercules, USA). Briefly, the RNA samples and oligo (dT) primers were mixed and denatured at 70 °C for 10 min. The tubes were then immediately placed on ice for at least 1 min. The transcription mixture and the RNase inhibitor were added, and the mixture was then incubated at 37 °C for 5 min. The first-strand cDNA synthesis was initiated after the addition of the Moloney Murine Leukemia Virus (M-MuLV) Reverse Transcriptase (ThermoFisher scientific, CA, USA), and the reverse-transcriptase reaction was performed at 42 °C for 1 h. Lastly, the enzyme was inactivated at 70 °C for 10 min. The reaction was performed in triplicate to reduce any reverse-transcription efficiency differences. The cDNAs were stored at −80 °C and diluted to 1:5 with RNase-free water for their use as a template in the real-time PCR analysis.

### Real-time RT-PCR (TaqMan™) analysis

The real-time RT-PCR analysis was conducted using the ABI7900HT machine (Applied Biosystems). All of the TaqMan RT-PCR reagents, including the primers and probes, were purchased from Applied Biosystems. The TaqMan analysis was conducted using predesigned and optimized Assays on Demand (Applied Biosystems). The following assays were used: HIF-1α (ID: Hs00936371_m1), HIF-2α (ID: Hs01026149_m1), OCT4 (ID: Hs03005111_g1), NANOG (ID: Hs02387400_g1), SOX2 (ID: Hs01053049_s1), REX1 (ID: Hs01938187_s1), MIF (ID: Hs00236988_g1), KLF2 (Hs00360439_g1), PPAR γ (ID: Hs01115729_m1), and GAPDH (ID: Hs00266705_g1). The reaction parameters are 2 min at 50 °C hold, 30 min at 60 °C hold, and 5 min at 95 °C hold, and this was followed by 45 cycles of 20 s at 94 °C for a melting, and 1 min at 60 °C for an annealing/extension. All of the measurements were performed in duplicate or triplicate. The results were analyzed using the ABI sequence-detection software version 2.0 (Applied Biosystems). A relative quantitation was conducted using the GAPDH as a reference gene, and it was validated using the NormFinder software. Since all of the used assays were optimized for PCR efficiency by the manufacturer, the mRNA-expression levels were estimated according to the delta-Ct values.

### Luciferase-reporter assay

To assay for the AP-1-, NF-κB-, CRE-, and HRE-promoter activities, the hAMSCs were transfected with AP-1 (Stratagene, La Jolla, CA, USA), NF-κB (Stratagene, La Jolla, CA, USA), CRE-Luc (Stratagene, La Jolla, CA, USA), or HRE-Luc reporters (Addgene, MA, USA), along with 1 μg of the Renilla-luciferase expression vector that was driven by a thymidine-kinase promoter (Promega, Madison, WI, USA) (internal standard) using the DharmaFECT^®^ Duo transfection reagent (Thermo Fisher Scientific, Inc., Waltham, MA, USA) according to the manufacturer’s protocols. At 24 h post-transfection, the cells were cultured in the MesenPRO RS^TM^ medium for 24 h and then irradiated with the indicated doses of UVA. Following the UVA irradiation, the cells were immediately incubated with the indicated concentrations of sinapic acid (Sigma–Aldrich, St. Louis, MO, USA) in the presence or absence of forskolin (Tocris, Bristol, UK), phorbol myristate acetate (PMA) (Sigma–Aldrich, St. Louis, MO, USA), tumor-necrosis factor-α (TNF-α) (Sigma–Aldrich, St. Louis, MO, USA), PD98059 (Cell Signaling Technology, Beverly, MA, USA), SB203580 (Cell Signaling Technology, Beverly, MA, USA), SP600125 (Cell Signaling Technology, Beverly, MA, USA), pyrrolidine dithiocarbamate (PDTC) (Sigma–Aldrich, St. Louis, MO, USA), or N-(2-[p-Bromocinnamylamino]ethyl)-5-isoquinolinesulfonamide (H89) (Sigma–Aldrich, St. Louis, MO, USA) for 24 h. The cells were then harvested, lysed, and centrifuged. Next, the supernatants were assayed for luciferase activity using the Dual Luciferase Assay system (Promega, WI, USA) on the LB953 luminometer (Berthold, Germany). The activities were expressed as a ratio of the AP-1-, NF-κB-, or CRE-dependent firefly-luciferase activity to the thymidine-kinase Renilla-luciferase activity (% control) that served as a control. The results were confirmed with eight independent transfections.

### Immunoblotting

The hAMSCs were irradiated with the indicated doses of UVA and then incubated with the indicated concentrations of sinapic acid for the indicated time under serum-free conditions or under adipogenic condition. The cells were washed twice with cold PBS (Sigma–Aldrich, St. Louis, MO, USA), followed by a lysing in 150 μl of sample buffer (100 mM Tris-HCl; Sigma–Aldrich, St. Louis, MO, USA) with a pH of 6.8, 10% glycerol (Sigma–Aldrich, St. Louis, MO, USA), 4% sodium dodecyl sulfate (SDS) (Sigma–Aldrich, St. Louis, MO, USA), 1% bromophenol blue (Sigma–Aldrich, St. Louis, MO, USA), and 10% β-mercaptoethanol (Sigma–Aldrich, St. Louis, MO, USA). Next, the samples were resolved using sodium dodecyl sulfate-polyacrylamide gel electrophoresis (SDS-PAGE) and then transferred to Immobilon-P PVDF membranes (Millipore Corporation, Bedford, MA, U.S.A.). These membranes were incubated overnight at 4 °C with an anti-HIF-1α antibody (Novus Biologicals, Beverly, MA, USA), anti-HIF-2α antibody (Novus Biologicals, Beverly, MA, USA), anti-phospho-AMPKα antibody (Thr172) (Cell Signaling Technology, Inc., Beverly, MA, USA), anti-AMPKααantibody (Cell Signaling Technology), and anti-β-actin antibody (Sigma–Aldrich, St. Louis, MO, USA). The membranes were subsequently washed three times with a Tris-buffered saline containing the Tween-20 (TBST; Sigma–Aldrich, St. Louis, MO, USA), probed with a horseradish peroxidase-conjugated secondary antibody (Sigma–Aldrich, St. Louis, MO, USA), and developed using an enhanced chemiluminescence (ECL) western-blot detection system (Amersham Biosciences).

### MAPK-phosphorylation analysis

The levels of phospho-SAPK/JNK (Thr183/Tyr185), phospho-p38 MAPK (Thr180/Tyr182), JNK, and p38 MAPK were measured using the PathScan Inflammation Multi-Target Sandwich ELISA Kit (Cell Signaling Technology) according to the manufacturer’s instructions. The levels of the phospho-p42/44 MAPK (Thr202/Tyr204) and p42/44 MAPK expressions were also determined using the PathScan Cell Growth Multi-Target Sandwich ELISA Kit (Cell Signaling Technology) according to the manufacturer’s instructions.

### ELISA

The hAMSCs were irradiated with the indicated doses of UVA and then incubated with the indicated concentrations of sinapic acid for the indicated time under serum-free conditions or under adipogenic condition. After the incubation, the concentration of PGE_2_, cAMP, and MIF in the culture supernatant was measured using an ELISA kit for PGE_2_ and cAMP (ENZO Life Sciences International, Inc., PA, USA) and an ELISA kit for MIF (Genzyme, Minneapolis, MN, USA). The culture supernatants were added into 96-well plates. Alkaline phosphatase-conjugated PGE_2_ or cAMP and either the antibodies of PGE_2_ or cAMP were added to sample wells and incubated at room temperature for 2 h. The sample wells were then washed with PBS, followed by the addition of a p-nitrophenyl phosphate (pNpp)-substrate solution. Lastly, the samples were incubated at room temperature for 1 h, and their absorbance values were read according to the manufacturer’s instructions.

### Statistical analysis

All of the data are expressed as mean ± SD. The comparison between the control and the treated group was performed using the one way analysis of variance (ANOVA), and it was followed by the Tukey’s multiple-comparison test for which the GraphPad Prism (5.0) (GraphPad, La Jolla, CA, USA) was used. Statistical significance was considered when the *p* value is less than 0.05.
